# Supplementary material for: Venomix: a simple bioinformatic pipeline for identifying and characterizing toxin gene candidates from transcriptomic data
Source: PeerJ. 2018 Jul 31;6:e5361. doi: 10.7717/peerj.5361 (PMC6074769; doi:10.7717/peerj.5361)
Supplement: Supplemental Information 3 [file peerj-06-5361-s003.gz › FinalOutput_E-6/Cystatin_2/finaltree.pdf]

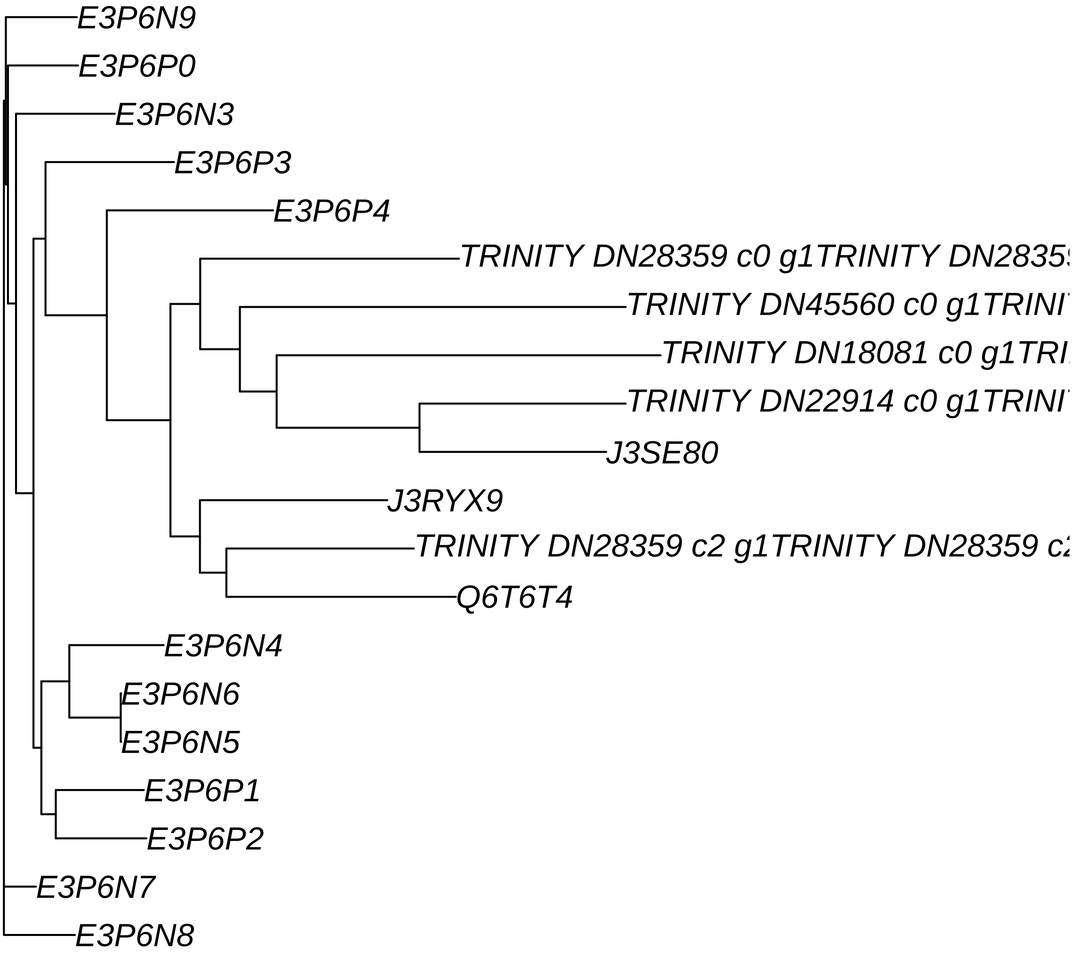
E3P6N9
E3P6P0
E3P6N3
E3P6P3
E3P6P4
TRINITY DN28359 c0 g1TRINITY DN2835
TRINITY DN45560 c0 g1TRINI
TRINITY DN18081 c0 g1TRI
TRINITY DN22914 c0 g1TRINI
J3SE80
J3RYX9
TRINITY DN28359 c2 g1TRINITY DN28359 c
Q6T6T4
E3P6N4
E3P6N6
E3P6N5
E3P6P1
E3P6P2
E3P6N7
E3P6N8
